# Association Between the Simplest Clinical Factors and Emergency Department Dispositions: A Retrospective Observational Study

**DOI:** 10.7759/cureus.12844

**Published:** 2021-01-21

**Authors:** Kasumi Satoh, Manabu Okuyama, Hajime Nakae

**Affiliations:** 1 Department of Emergency and Critical Care Medicine, Akita University Graduate School of Medicine, Akita, JPN

**Keywords:** emergency department, epidemiology, decision support techniques

## Abstract

The emergency department (ED) is a complex and busy environment that requires rapid decision making. We assessed the relationship between disposition from the ED and information that can be obtained at a glance in the ED. The presentation of the implications of commonplace information could assist healthcare providers in ensuring smooth and safe ED care. Thus, we aimed to quantitatively assess how readily obtainable findings, such as age, sex, and vital signs, are involved in the disposition of adult patients transferred to the ED. This retrospective observational study was conducted in the ED of a regional university hospital containing approximately 600 beds. Of the 685 patients included in the analysis, 351 patients were admitted to the hospital (including 12 deaths in the ED) and 334 patients were discharged from the ED. A multiple logistic regression model that included age, sex, systolic blood pressure, heart rate, respiration rate, temperature, and SpO_2_ as variables identified independent associations between age (p=0.003), sex (p<0.001), systolic blood pressure (p=0.023), heart rate (p<0.001), and respiratory rate (p=0.028) and admission from the ED. The receiver operating characteristic curves drawn from the multiple logistic regression model comprising these five variables had an area under the curve (AUC) of 0.701 (95% confidence interval: 0.657-0.744, p<0.001). Examination of sensitivity, specificity, and likelihood ratios (LRs) for these five variables for clinical utility showed a slightly higher sensitivity for age ≥50 years (0.849) and respiratory rate ≥18 bpm (0.769); higher specificity for systolic blood pressure ≤100 mmHg (0.938), pulse rate ≥100 bpm (0.834), and respiratory rate ≥26 bpm (0.887); higher positive LR for systolic blood pressure ≤100 mmHg (2.039) and pulse rate ≥110 bpm (2.729); and slightly lower negative LR for age ≥50 years (0.656), male sex (0.647), respiratory rate ≥20 bpm (0.669). These results are meaningful as they quantify the intuition of a skilled clinician, which can help in clinical decision making, reduce errors, and promote clinical education. Our study provides a basis for explaining to novice healthcare providers that the careful observation of ED patients, even in the absence of special laboratory tests, can help them to make judgments regarding the disposition of the patients from the ED. In conclusion, age, sex, systolic blood pressure, heart rate, and respiratory rate were independently associated with a disposition from the ED. A multivariate model including these five variables showed the moderate-quality potential to predict admission from the ED. The sensitivity, specificity, and LR of systolic blood pressure, heart rate, and respiratory rate showed the characteristics of each vital sign. These provide healthcare providers in the ED an immediate clue regarding the patient’s illness.

## Introduction

In the emergency department (ED), decisions are made rapidly in a complex and busy environment. Decisions regarding a patient’s disposition are especially difficult in this setting. Several attempts have been made to predict admission from the ED; however, there is a trade-off between simplicity and accuracy [1]. To improve accuracy, existing predictors have included items that cannot be obtained at a glance, such as elaborate community-specific scales and chronic comorbidities [1-3]. Thus, no solid predictor of admission from the ED has been widely applied in the clinical setting. Some of the existing predictors are for bed control purposes; that is, the early alerting of wards to the presence of patients who are indicated for disposition rather than to support clinical decision making [1,3].

In contrast, this study assessed the relationship between disposition from the ED and information that can be obtained at a glance in the ED. The presentation of the implications of commonplace information could assist healthcare providers in ensuring smooth and safe ED care. In addition, this information is educational as it reveals important points to consider when examining ED patients.

Thus, the current study aimed to quantitatively assess how readily obtainable findings, such as age, sex, and vital signs, are involved in the disposition of patients transferred to the ED.

## Materials and methods

Study setting and participants

This retrospective observational study was conducted in the ED of a regional university hospital with approximately 600 beds. Our ED is an advanced medical facility that receives approximately 1600 ambulances per year.

The eligibility criteria were patients aged >18 years who were transported to the ED. We analyzed a database in which the age, sex, and vital signs were recorded on the arrival of patients to the ED. The analysis included data from July 1, 2018 to February 28, 2019. The Ethics Committee of Akita University School of Medicine approved this retrospective study (Approval number/ID 2283). The need for informed consent was waived due to the observational nature of the study.

Outcome and variables

The outcome of this study was the admission from the ED. All patients who were transferred to a general ward or the intensive care unit (ICU) and those who died in the ED were considered cases of admission from the ED. Patients who were discharged from the ED were not considered.

The database included information on the method of arrival (ambulance or helicopter), age, sex, systolic blood pressure, diastolic blood pressure, respiratory rate, heart rate, temperature, blood oxygen saturation, and disposition of patients transported to the ED. The vital signs in the database were measured at the time of arrival in the ED. The disposition included discharge from the ED, admission to the general ward, admission to the ICU, and death in the ED. All the information contained in the database was included in the analysis. The first choice of vital sign collection methods are the non-invasive measurement of the blood pressure at the upper extremity, anterior thoracic three-point monitor electrocardiogram to determine the heart rate, axillary temperature for body temperature, visual measurement of respiratory rate by the ED nurse, and percutaneous oxygen saturation (SpO2) for oxygen saturation. The database did not contain diagnostic information, treatment information, or laboratory data. Patient age and sex were automatically retrieved from the electronic medical records; other information was manually recorded by the ED nurse at each patient visit.

Statistical methods

Differences between cases of admission from ED and discharge from ED were assessed using the Wilcoxon rank-sum test for continuous variables and the chi-square test for categorical variables. Multivariate logistic regression analyses were performed by adjusted for age, sex, and vital signs. Since the systolic and diastolic blood pressures were correlated (Spearman’s rank correlation coefficient, r=0.7696; p<0.001), only systolic blood pressure was included. A receiver operating characteristic (ROC) curve was drawn from the model using variables that were significant in the previous multivariate logistic analysis and the area under the ROC curve (AUC) was calculated to examine the fit in predicting hospitalization from the ED.

The sensitivity (Se), specificity (Sp), positive likelihood ratio (LR+), and negative likelihood ratio (LR-) for the outcome were calculated for the variables that were significant in the multivariate logistic analysis. The cutoff values for each variable were examined while conducting sensitivity analysis among the abnormal values. Age was analyzed every five years from 50 to 80 years, hypotension was analyzed every 10 mmHg from 80 to 110 mmHg for systolic blood pressure, hypertension was analyzed every 10 mmHg from 140 to 220 mmHg for systolic blood pressure, pulse rate was analyzed every 10 bpm from 80 to 120 bpm, and respiratory rate was analyzed every 2 bpm from 18 to 28 bpm. Missing data were not imputed.

Statistical analyses were conducted using Stata® software, version 15.1 (StataCorp, College Station, Texas, USA). Significance was defined as a two-sided p-value of <0.05.

## Results

**Figure 1:**
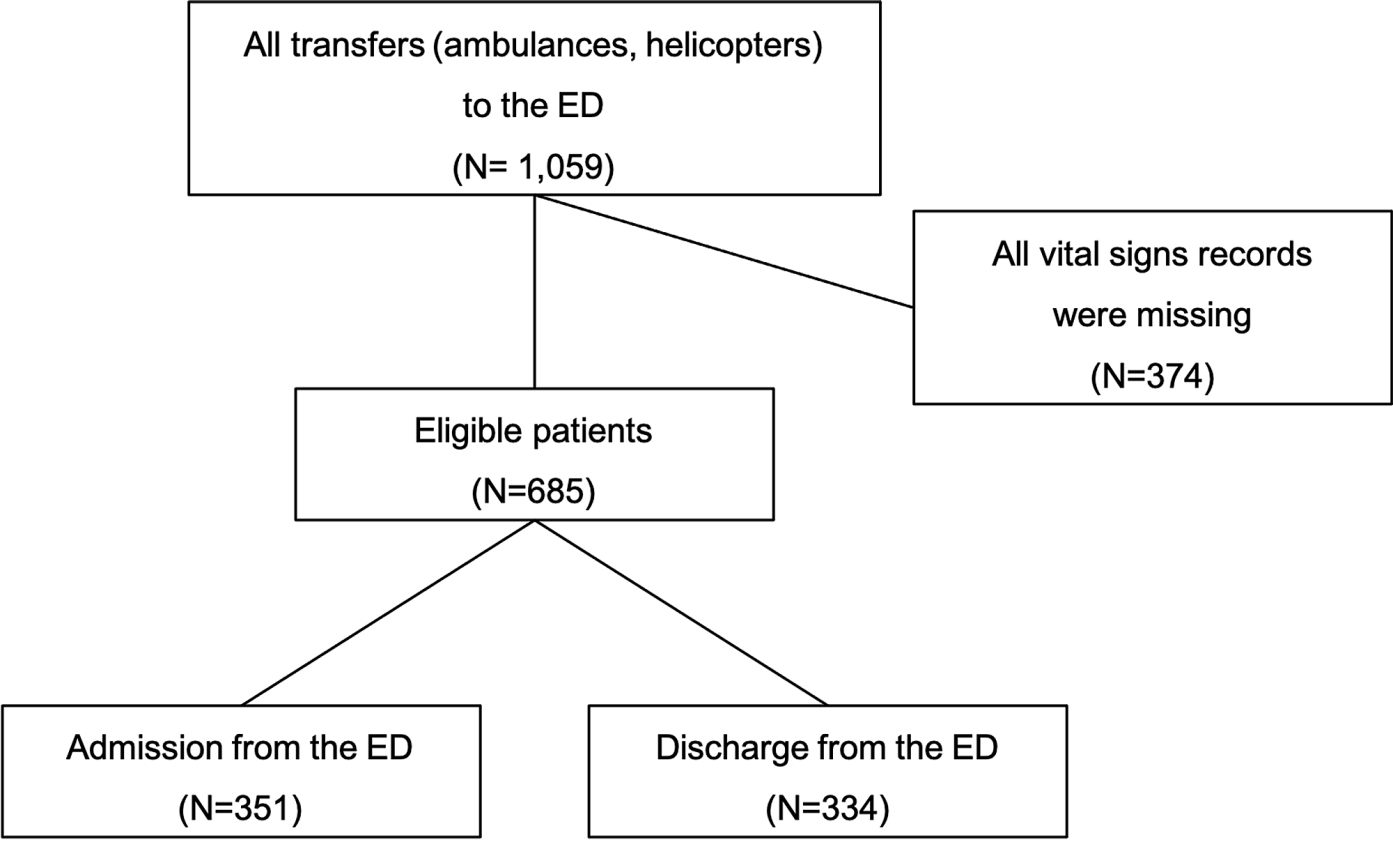
Patient Flow Diagram Of the 1,059 eligible patients who were transported to the ED, patients for whom vital signs were not recorded were excluded from the analysis. All patients who were transferred to a general ward or the intensive care unit (ICU) from the ED and those who died in the ED were considered cases of admission from the ED. Of the 685 patients who were included in the analysis, 351 patients were admitted to the hospital and 334 patients were discharged from the ED.

Of the 1,059 eligible patients, patients for whom vital signs were not recorded were excluded from the analysis. These patients included those who used an ambulance to transfer to our ward from another hospital but who were not treated in the ED, those who had data entry errors in nurses’ database, and those admitted to the ED for cardiopulmonary arrest. Of the 685 patients who were included in the analysis, 351 patients were admitted to the hospital (including 12 deaths in the ED) and 334 patients were discharged from the ED (Figure 1). Among the eligible patients, 69 patients had missing data on systolic blood pressure, 79 patients had missing data on diastolic blood pressure, 50 patients had missing data on heart rate, 99 patients had missing data on respiratory rate, 30 patients had missing data on temperature, and 65 patients had missing data on SpO_2_.

The baseline characteristics of the patients are shown in Table 1. The median patient age was 69 years, and 56.87% of patients were male. The group admitted to the hospital had a significantly higher proportion of male patients, higher heart rate, higher respiratory rate, and higher body temperature than the group discharged from the ED.

**Table 1:** Baseline characteristics of the patients Data are expressed as medians (interquartile ranges) for continuous variables and numbers (%) for categorical variables. The differences between the admission and discharge groups from the ED were analyzed. The Wilcoxon rank-sum test was used for the continuous variables, and the chi-square test was used for categorical variables. Missing data were not included. bpm, beats per minute or breathes per minute; ED, emergency department.

	Variables		All (N=685)		Admit (N=351)		Discharge (N=334)		P-value
	Age	yr	69	(56-80)	69	(59-81)	68	(52-80)	0.139
	Male sex		369	(56.87%)	222	(63.25%)	147	(44.01%)	<0.001
Arrival means	Ambulance		668	(97.52%)	335	(95.44%)	333	(99.70%)	<0.001
	Helicopter		17	(2.48%)	16	(4.56%)	1	(0.30%)	
Vital signs	Systolic blood pressure	mmHg	140	(121-161)	138	(120-159)	141	(121-163)	0.101
	Diastolic blood pressure	mmHg	81	(70-93)	80	(68-93)	81	(71-93)	0.115
	Heart rate	bpm	83	(72-98)	88	(74-102)	81	(70-92)	<0.001
	Respiratory rate	bpm	20	(17-24)	20	(18-25)	19	(16.5-22)	0.004
	Body temperature	℃	36.7	(36.4-37.0)	36.7	(36.4-37.2)	36.6	(36.4-36.9)	0.012
	Oxygen saturation	%	98.0	(96.5-100.0)	98	(96-100)	98	(97-99)	0.740
Type of ward	General ward		213	(31.09%)	-		-		-
	Intensive care unit		126	(18.39%)	-		-		-
	Death at ED		12	(1.75%)	-		-		-

**Figure 2:**
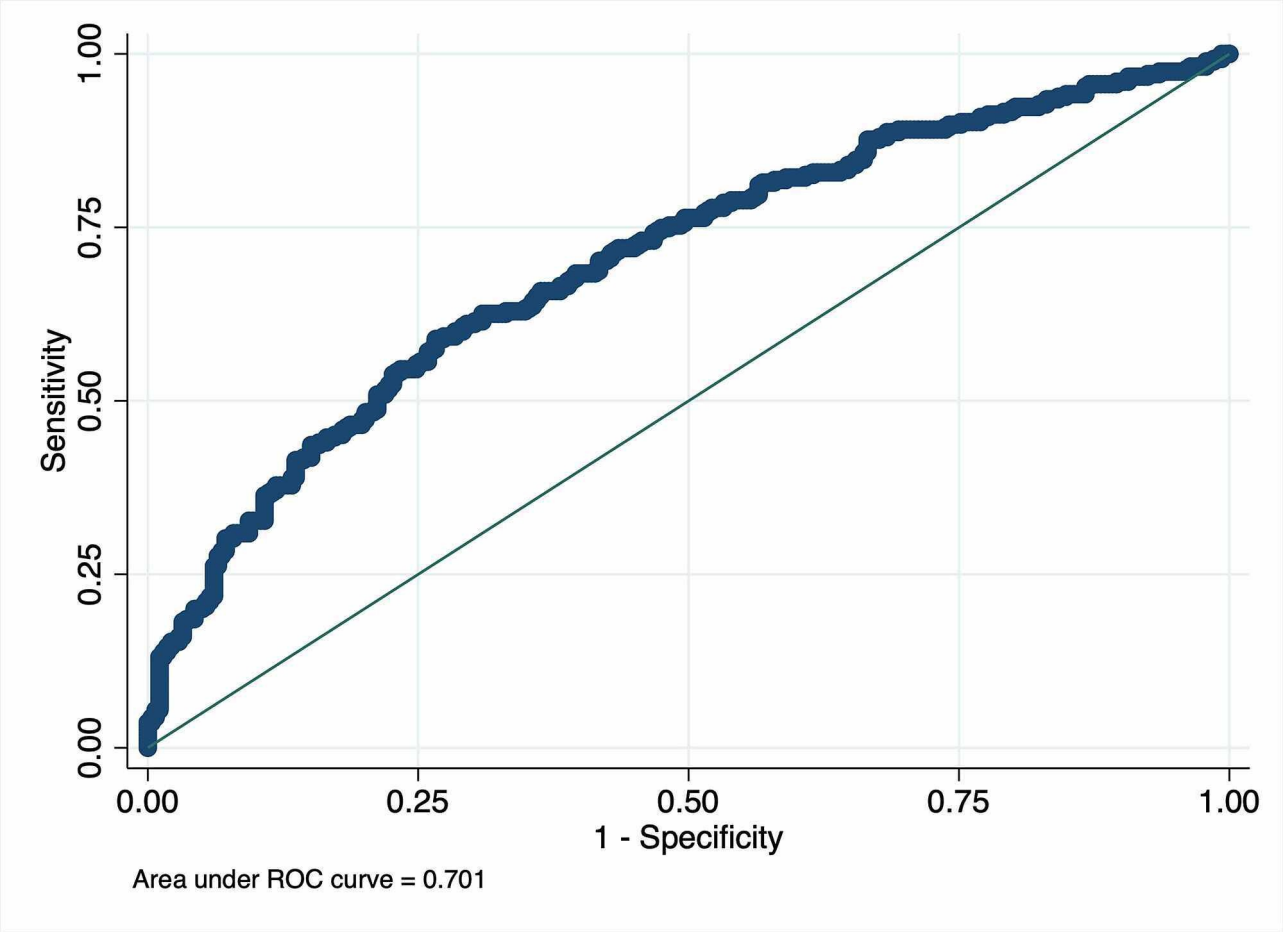
The receiver operating characteristic curves drawn from the multiple logistic regression model including variables independently associated with admissions from the emergency department. The area under the receiver operating characteristic (ROC) curves was 0.701 (95% confidence interval: 0.657–0.744, p<0.001).

In a multiple logistic regression model that included age, sex, systolic blood pressure, heart rate, respiration rate, temperature, and SpO2 as variables, we identified independent associations between age (p=0.003), sex (p<0.001), systolic blood pressure (p=0.023), heart rate (p<0.001), and respiratory rate (p=0.028) and hospitalization from the ED (Table 2). The ROC curves drawn from the multiple logistic regression model including these five variables (Table 3) are shown in Figure 2. The model showed discriminatory power for predicting hospital admissions, with an AUC of 0.701 (95% confidence interval: 0.657-0.744, p<0.001).

**Table 2:** Factors influencing patients' disposition from the emergency department in multivariable logistic regression analysis. ORs (95% CI) were estimated using multivariate analysis adjusted for age, sex, and vital signs. Due to collinearity with the diastolic blood pressure, the systolic blood pressure was used for all analyses. OR, odds ratio; CI, confidence interval; bpm, beats per minute or breathes per minute.

Multivariate logistic regression analysis (N=526)					
		OR	95% CI		P-value
Age	yr	0.017	0.006	0.027	0.003
Male sex		0.808	0.440	1.176	<0.001
Systolic blood pressure	mmHg	-0.007	-0.013	-0.001	0.023
Heart rate	bpm	0.019	0.009	0.029	<0.001
Respiratory rate	bpm	0.038	0.004	0.072	0.028
Body temperature	℃	0.208	-0.030	0.446	0.086
Oxygen saturation	%	-0.024	-0.093	0.046	0.504

**Table 3:** The multiple logistic regression model that includes only five factors independently associated with the patients' disposition from the ED. ORs (95% CI) were estimated using multivariate analysis adjusted for age, sex, systolic blood pressure, heart rate, and respiratory rate. The variables for adjustment were five factors that were independently associated with patients' disposition from the ED. OR, odds ratio; CI, confidence interval; bpm, beats per minute or breathes per minute.

Multivariate logistic regression analysis (N=553)					
		OR	95% CI		P-value
Age	yr	0.018	0.008	0.029	0.001
Male sex		0.827	0.470	1.185	<0.001
Systolic blood pressure	mmHg	-0.007	-0.013	-0.001	0.018
Heart rate	bpm	0.022	0.012	0.031	<0.001
Respiratory rate	bpm	0.045	0.012	0.078	0.007

The Se, Sp, LR+, and LR- for admission from the ED for the variables that were significant in the multiple logistic analysis are shown in Table 4. Among all the variables assessed in this study, the highest Se (0.849) was observed when age ≥50 years was used as a cutoff. Among the vital signs, the highest Se (0.769) was obtained when a respiratory rate of ≥18 bpm was used as the cutoff. The Sp values were relatively high when systolic blood pressure ≤ 100 mmHg (Sp 0.938) and pulse rate ≥ 100 bpm (Sp 0.834) were used as the cutoff. Regarding respiratory rate, a high Sp (0.887) was observed for a cutoff of ≥26 bpm. The LR+ was high when systolic blood pressure ≤100 mmHg (LR+ 2.039) and pulse rate ≥110 bpm (LR+ 2.729) were used as the cutoff. We observed no significant values for the LR-; however, it was relatively low for age ≥50 years (LR- 0.647), male sex (LR- 0.656), and respiratory rate ≥20 bpm (LR- 0.669). For hypertension, no cutoffs showed significant differences or constant trend.

**Table 4:** The sensitivity, specificity, and likelihood ratios for admission from the emergency department (ED) for variables that were significantly associated with admission from the ED in the multiple logistic analysis. bpm, beats per minute or breathes per minute.

			Sensitivity	Specificity	LR+	LR-	P-value
Sex		Male	0.632	0.560	1.437	0.656	<0.001
Age	yr	≧50	0.849	0.234	1.108	0.647	0.006
		≧60	0.749	0.326	1.112	0.768	0.029
		≧70	0.481	0.524	1.011	0.990	0.887
		≧80	0.279	0.731	1.036	0.987	0.775
Systolic pressure	mmHg	≦110	0.175	0.866	1.309	0.952	0.157
		≦100	0.126	0.938	2.039	0.931	0.006
		≦90	0.071	0.984	4.372	0.944	0.001
		≦80	0.045	0.997	13.909	0.958	0.001
Heart rate	bpm	≧90	0.442	0.713	1.543	0.782	<0.001
		≧100	0.287	0.834	1.731	0.855	<0.001
		≧110	0.165	0.939	2.729	0.889	<0.001
		≧120	0.109	0.978	4.891	0.911	<0.001
Respiratory rate	bpm	≧18	0.769	0.318	1.128	0.726	0.018
		≧20	0.656	0.514	1.350	0.669	<0.001
		≧22	0.422	0.688	1.353	0.840	0.006
		≧24	0.333	0.774	1.475	0.861	0.004
		≧26	0.190	0.887	1.685	0.913	0.009
		≧28	0.143	0.914	1.669	0.937	0.030

## Discussion

The results of this study showed that age, sex, systolic blood pressure, heart rate, and respiratory rate were independently associated with the disposition of patients from the ED. A multivariate model comprising these five variables predicted moderate-quality potential to predict admission from the ED. In addition, examination of the Se, Sp, and LR for these five variables for clinical utility showed slightly higher sensitivity for age ≥50 years and respiratory rate ≥ 18 bpm; higher specificity for systolic blood pressure ≤ 100 mmHg, pulse rate ≥ 100 bpm, and respiratory rate ≥ 26 bpm; higher LR+ for systolic blood pressure ≤ 100 mmHg and pulse rate ≥ 110 bpm; and slightly lower LR- for age ≥50 years, male sex, and respiratory rate ≥ 20 bpm.

In this study, age, sex, systolic blood pressure, heart rate, and respiratory rate were independently associated with the disposition from the ED. Although some studies have also noted that elderly patients are more likely to be admitted from the ED [1-3], to our knowledge, no study has reported male sex is independently associated with admission from the ED. Male sex contributes to the risk of cardiovascular disease, with a significantly higher risk of cardiovascular events such as death, myocardial infarction, stroke, and heart failure hospitalization [4]. In sepsis, male sex has been reported as a disadvantage related to illness severity and life prognosis [5,6]. These previous findings are consistent with our finding of an increased risk of ED admission in men. Blood pressure, respiratory rate, and pulse rate were identified as significant vital signs in the current study, which is similar to the findings reported by Franklin et al. [7] who indicated that these vital signs are well-known warning signs seen within six hours of in-hospital cardiac arrest. In both ED patients and inpatients, the evaluation points for recognizing illness may be similar.

Further, in this study, a multivariate model comprising five variables independently associated with admission from the ED showed moderate-quality potential to predict admission from the ED (AUC 0.701). Compared to previous studies, the current study used the simplest variables from the essential information available when a patient is transferred to the ED. The variables we have adopted have the advantage of easy availability, with only blood pressure measurement requiring special medical equipment. Our variables are nonspecific and, therefore, comparatively less sophisticated in terms of accuracy. Paker et al. proposed a model for predicting hospitalization from the ED that considered age, race, place of residence, visit the day of the week, visit time, Singapore triage category (based on patients’ symptoms and vital signs), method of arriving at the hospital, and fever status, which showed an AUC of 0.825 [1]. Cameron et al. also proposed a model for predicting admission from the ED that considered the Manchester triage category (based on patients’ symptoms and vital signs), age, National Early Warning Score (containing vital signs classification), means of the hospital visit, referral hospital status, and history of hospitalization in the past year, with an AUC 0.874 [2]. Although these models are highly accurate in predicting hospital admissions from the ED, they contain complex and region-specific scoring systems that are not practical in daily practice. Thus, they are not intended for clinical decision making [1]. Our results do not suggest an accurate predictive model for admission from the ED; however, they suggest that healthcare providers should carefully assess patients in the ED in an actual clinical setting, which can support prompt decision making.

Furthermore, we evaluated the Se, Sp, and LRs for the five variables independently associated with admission from the ED (age, sex, systolic blood pressure, heart rate, and respiratory rate) to quantify their clinical utility. Regarding age, the Se was high for age ≥50 years (Se 0.849), suggesting that patients aged <50 years were less likely to be admitted from the ED. Furthermore, older age did not necessarily mean a higher tendency for admission from the ED. Regarding systolic blood pressure, the Sp increased from ≤100 mmHg (Sp 0.938) and was more marked with a lower cutoff. The same trend was observed for LR+, increasing for ≤100 mmHg (LR+ 2.039). In patients with systolic blood pressure ≤100 mmHg, healthcare providers should be aware of the potential for the patient to be admitted from the ED. Regarding heart rate, the Sp increased from ≥100 bpm (Sp 0.834) and further increased at higher cutoffs. The same trend was observed for LR+, increasing from ≥110 bpm (LR+ 2.729). A heart rate of ≥120 bpm showed an Sp of 0.978 and an LR+ of 4.891, strongly suggesting admission from the ED. Similar to systolic blood pressure, a pulse rate of 100 was also a warning sign. Thus, a clinical decision to discharge a patient with a heart rate ≥120 bpm from the ED should be re-evaluated. The respiratory rate tended to slightly differ from the other vital signs. A respiratory rate of ≥18 bpm showed a relatively, slightly high Se of 0.769 and a respiratory rate of ≥20 bpm showed a relatively low LR- of 0.669. Neither systolic blood pressure nor heart rate had any meaningful Se or LR-; therefore, the implications of respiratory rate differ from those of other vital signs. This may reflect the finding that tachypnea is observed in almost all conditions of clinical decline before the detection of abnormalities in other vital signs [8]. The Sp was also higher when the respiratory rate was ≥26 bpm (0.887) in the present study. It is important to focus on a respiratory rate of less than 18-20 bpm or more than 26 bpm. These results are meaningful as they quantify the intuition of a skilled clinician, which can help in clinical decision making, reduce errors, and promote clinical education. Although all healthcare providers involved in the ED should be aware of the characteristics associated with admission from the ED, this is not necessarily accomplished. For instance, respiratory rate recordings were skipped for about 15% of eligible participants in the current study; however, given the importance of respiratory rate, healthcare providers should not omit this measurement. Despite the clinical significance of abnormalities in respiratory rate, tachypnea is easily overlooked [8]. Respiratory rate was the most neglected factor recorded in the current study.

Our study is meaningful as it provides a key assessment of the moment a healthcare provider encounters a patient in the ED. In particular, our study provides a basis for explaining to novice healthcare providers that the careful observation of ED patients, even in the absence of special laboratory tests, can help them to make judgments regarding the disposition of the patients from the ED.

This study has several limitations. First, because patient populations and indications for admission vary between hospitals, the external validity of our findings requires testing in EDs of different hospitals. Second, our results have moderate predictive quality for clinical application in isolation. However, in the real-world setting, clinicians combine symptoms, physical findings, laboratory data, and social context to determine patient dispositions; hence, our results can assist clinicians. Third, our database does not include information on pre-existing diseases, treatment, diagnosis, or length of stay in the ED, because this study was designed to evaluate essential patient information that can be obtained at a glance in ED practice. However, additional patient information could detail the characteristics of the ED practices in the study and provide further insight related to patient dispositions. In the future, we intend to build a database that includes information such as treatment and diagnosis to improve the quality of the study. Finally, vital signs may have missing values and contain recorder bias; for instance, the data may not have been recorded because the patient was clearly very ill or not ill.

## Conclusions

In conclusion, age, sex, systolic blood pressure, heart rate, and respiratory rate were independently associated with the disposition from the ED. A multivariate model including these five variables showed the moderate-quality potential to predict admission from the ED. The Se, Sp, and LR of systolic blood pressure, heart rate, and respiratory rate showed the characteristics of each vital sign. These provide healthcare providers in the ED an immediate clue as to the patient’s illness.
